# *In Vitro* Evaluation of Recombinant Bone Morphogenetic Protein-2 Bioactivity for Regenerative Medicine

**DOI:** 10.1089/ten.tec.2019.0156

**Published:** 2019-09-17

**Authors:** Stephanie L. Fung, Xiaohuan Wu, Julian P. Maceren, Yong Mao, Joachim Kohn

**Affiliations:** New Jersey Center for Biomaterials, Rutgers—The State University of New Jersey, Piscataway, New Jersey.

**Keywords:** BMP-2, bioactivity, alkaline phosphatase activity, bone regeneration

## Abstract

Recombinant human bone morphogenetic protein-2 (rhBMP-2) is a commonly used growth factor in bone regeneration due to its high potency and ability to induce osteogenic differentiation of osteoblasts and osteoblast precursors. When designing delivery systems for rhBMP-2, the activity of the loaded and released protein is an important consideration. The variability in the experimental design parameters used to measure rhBMP-2 activity *in vitro* has precluded comparative analysis. Here, for the first time, we report a direct comparison of the assay parameters used in rhBMP-2 bioactivity assays in the literature and an evaluation of commercially available rhBMP-2 obtained from different vendors. Most published rhBMP-2 assays use W-20-17 (mouse stromal), MC3T3 (preosteoblast), or C2C12 (myoblast) cell lines. We found that each model cell line has an optimal concentration range over which it is most sensitive to rhBMP-2 induction. Therefore, it is difficult to find one single bioassay protocol that could be universally used. In addition, we established a correlation between protein concentration (as measured by enzyme-linked immunosorbent assay) and protein activity (as measured by alkaline phosphatase induction). We found that the expression system used to produce the rhBMP-2 had the greatest effect on its activity and stability *in vitro*. Establishing a standard method of measuring rhBMP-2 activity *in vitro* is the first step toward developing an *in vitro–in vivo* correlation between measured activity and clinical outcomes.

**Impact Statement:**

This work is a systematic evaluation of the experimental parameters of the most widely used *in vitro* recombinant human bone morphogenetic protein-2 (rhBMP-2) activity assays. The variations in assays reported in the literature have challenged the reproducibility and translation of work using rhBMP-2 as a bone-inducing growth factor. By elucidating the effect of model cell line on the dose-dependent alkaline phosphatase response to rhBMP-2 induction and by establishing a correlation between protein activity and protein concentration by enzyme-linked immunosorbent assay using commercially available rhBMP-2, this work is a significant step toward developing an *in vitro–in vivo* correlation between quantified activity and clinical efficacy.

## Introduction

Bone morphogenetic protein-2 (BMP-2) is a strong inducer of osteogenic differentiation.^1–4^ It is currently the only Food and Drug Administration (FDA)-approved osteoinductive factor used clinically for bone fusion applications in the United States.^5^ BMP-2 can be isolated from native bone, or more commonly, expressed as a recombinant human protein (rhBMP-2). The structure and function of the native human BMP-2 have been thoroughly addressed.^6^ Mature, bioactive BMP-2 molecules are in the form of a dimer composed of two monomeric units linked by disulfide bonds. Reduction of these disulfide bonds results in the complete loss of bioactivity.^6^ RhBMP-2 has been shown to increase alkaline phosphatase (ALP) expression through the Wnt signaling pathway in many cell types, making ALP expression a universal marker for measuring rhBMP-2 bioactivity *in vitro.*^7^

rhBMP-2 can be produced in various expression systems, including a mammalian source, such as the Chinese hamster ovary (CHO) or human embryonic kidney (HEK) cells; or a bacterial source, such as *Escherichia coli.*^7,8^ Recombinant proteins produced in mammalian cells are glycosylated in their final form, whereas those produced in bacterial cells do not undergo this post-translational modification.^8^

Although it has been reported that glycosylation is not essential for the biological activity of rhBMP-2,^9–11^ the glycosylation influences the biological function of the protein, especially in its effect on interactions between the protein and its carrier and on its distribution once delivered in the body. For example, it was shown that despite small differences in the isoelectric point of the *E. coli-* and CHO-derived rhBMP-2, the pharmacokinetics varied significantly *in vivo* due to the reduced solubility of nonglycosylated protein.^9^ In the case of rhBMP-2 mixed with a fibrin carrier, the reduced solubility of nonglycosylated rhBMP-2 improved the healing rate of critical-sized defects in a rat calvarial model.^11^

Different cell lines have been used to measure rhBMP-2 bioactivity *in vitro*. W-20-17 cells are derived from the bone marrow of a W^++^ mouse strain. These cells express low basal levels of ALP; however, upon induction with >6 ng/mL rhBMP-2 for 24 h, cells express higher than basal levels of ALP in a dose-dependent manner without an effect on cell proliferation.^12^ The W-20-17 cell line has been used to determine the bioactivity of rhBMP-2 delivered in hydrogels and microspheres,^10,13,14^ and is also the cell line used in the American Society for Testing and Materials (ASTM) Standard Test Method for *In vitro* Biological Activity of rhBMP-2 (F2131-02).

rhBMP-2 also has the ability to redirect C2C12 cells, a myoblast cell line, down the osteogenic lineage.^1^ ALP activity, osteocalcin production, and parathyroid hormone-induced 3′,5′-cAMP production were all upregulated upon incubation with >100 ng/mL rhBMP-2, which suggests emergence of an osteoblastic phenotype. These concentrations were also sufficient to inhibit myotube formation. Transforming growth factor beta-1 (TGF-β1) induction resulted in a decrease in osteocalcin production and ALP activity, which confirms the specificity of rhBMP-2 in converting myoblasts toward the osteoblastic lineage.^1^ Therefore, C2C12 cells are used as a model cell line to measure rhBMP-2 bioactivity in many studies.^15–18^ Other cells such as osteoblast progenitor cells (MC3T3) have also been used to determine the bioactivity of BMP-2.^19–21^

In this study, we evaluated and compared the sensitivity of the most widely used rhBMP-2 bioactivity assays. We explored the dose response of W-20-17, C2C12, and MC3T3 cells to the same batch of rhBMP-2 (*E. coli*-derived rhBMP-2). Next, we compared the bioactivity and stability of rhBMP-2 from different commercial sources. The results of our systematic study will help researchers to choose an appropriate bioactivity assay based on their research needs.

## Materials and Methods

### Cell culture

C2C12 cells were (ATCC CRL-1772) cultured according to manufacturer's instructions. Cells were maintained in Dulbecco's modified Eagle's medium (DMEM; Life Technologies) containing 10% fetal bovine serum (FBS; Atlanta Biologicals) and 35 μg/mL gentamicin (Sigma-Aldrich). Cells were passaged before reaching confluence, and medium was changed every 3–4 days. MC3T3-E1 cells (ATCC CRL-2593) were cultured in complete Alpha Minimum Essential Medium (αMEM; Life Technologies) containing 10% FBS and 35 μg/mL gentamicin. Cells were passaged upon reaching confluence, with media changes every 3–4 days.

W-20-17 cells (ATCC CRL-2623) were cultured according to the ASTM Standard Protocol (F2131-02). Basal medium was prepared by dissolving 13.3 g of DMEM with 4500 mg/L glucose and 4.0 mM l-glutamine (Sigma-Aldrich) and 2.226 g sodium bicarbonate (Sigma-Aldrich) in 800 mL of purified water. The pH was adjusted to 7.3 ± 0.1 using 0.2 N HCl, and the final volume was brought to 1 L. The basal medium was then sterile filtered through a 0.2 μm PES filter into sterile bottles, and then supplemented with heat-inactivated FBS (10%), l-glutamine (8 mM; Life Technologies), and gentamicin (50 μg/mL). Cells with the passage numbers between 3 and 6 were seeded at 2 × 10^5^ cells per T162 flask, and cultured for 4 days before seeding for bioactivity assays. All cells were cultured at 37°C, 5% CO_2_, and >95% humidity.

### BMP-2 sources

rhBMP-2 were obtained from East China University of Science and Technology (ECUST) or purchased from Peprotech, R&D Systems, or Humanzyme. The source information for the rhBMP-2 and international standard used is described in Table 1. To dissociate samples from their manufacturers, rhBMP-2 derived from CHO cells is denoted C1/C2/C3 in the text. HEK293-derived and *E. Coli*-derived rHBMP-2 are denoted H1 and E1/E2/E3, respectively. Proteins received as lyophilized powders were reconstituted according to manufacturer's instructions and stored at −80°C. Before analysis, concentrations of each stock solution were determined using a microbicinchoninic acid (BCA) kit (Thermo Fisher Scientific) according to manufacturer's instructions. Dilution of each sample was performed based on the concentration determined by BCA assay.

**Table 1.** Source Information for Commercially Available Recombinant Human Bone Morphogenetic Protein-2 and the International Standard

*Manufacturer*	*Source*	*Lot no.*
National Institute for Biological Standards and Control, Blanche Lane, United Kingdom	CHO cells	N/A
PeproTech, Inc., Rocky Hill, NJ	CHO cells	0215595
R & D System, Inc., Minneapolis, MN	CHO cells	MSA5216021
Medtronic, Minneapolis, MN	CHO cells	N/A
Humanzyme, Inc., Chicago, IL	HEK293 cells	0415-01
ECUST, Shanghai, China	*Escherichia coli*	N/A
R & D System, Inc.	*E. coli*	WN1415061
PeproTech, Inc.	*E. coli*	0614255

CHO, Chinese hamster ovary; ECUST, East China University of Science and Technology; N/A, not applicable.

### Bioactivity assays

The protocol for testing *in vitro* bioactivity of rhBMP-2 using C2C12 cells was adapted from the protocols described in the literature.^16^ Maintenance medium was prepared by adding FBS to a final concentration of 2% in DMEM (Life Technologies). C2C12 cells were plated at 1 × 10^4^ cells/well in a 96-well tissue culture-treated polystyrene plate (Denville Scientific, Inc.) and cultured in complete growth media (DMEM +10% FBS +35 μg/mL gentamicin) at 37°C for 24 h. rhBMP-2 was diluted to 1369 ng/mL in maintenance media, then serially diluted at 4.3-fold dilutions (unless otherwise noted) in a 96-well plate. Seven dilutions were prepared.

Growth medium was removed, the monolayer of cells was washed twice with sterile phosphate buffered saline (PBS), and 100 μL of maintenance media was added to each well. One hundred microliters of the maintenance media containing rhBMP-2 from the dilution series was added, resulting in the highest concentration of the series being 684.5 ng/mL. Cells were cultured in the absence of rhBMP-2 to determine background signal. Cells were incubated at 37°C, 5% CO_2_ for 72 ± 4 h unless otherwise noted. Medium was removed from all wells. Plate was washed with 200 μL PBS. Fifty microliters purified water was added to each well, and the plate was frozen at −80°C. Plates underwent two thaw-freeze cycles. The plate was brought to room temperature before development.

The assay mix was prepared by dissolving 170 mg p-nitrophenyl phosphate (PNPP) in 50 mL glycine buffer; the glycine buffer was prepared according to ASTM (F2131-02). Fifty microliters assay mix was added to each well, and the plate was incubated at room temperature on an orbital shaker. Measurements of absorbance at 405 nm were taken every 30 min on a Tecan Spark 10M plate reader (TECAN) until the highest reading of the plate was ∼1.0 optical density greater than the background signal. One hundred microliters 0.2 N NaOH was added to each well to stop the reaction. Absorbance was measured again at 405 nm. The dose response of MC3T3-E1 cells to rhBMP-2 induction was assayed using the same methods, with all culture carried out using αMEM in place of DMEM.

The protocol for testing *in vitro* bioactivity of rhBMP-2 using W-20-17 cells was adapted from the ASTM Standard (F2131-02). Assay medium was prepared by adding FBS (final concentration 10%), l-glutamine (final concentration 4 mM), and penicillin/streptomycin (Life Technologies, final concentration 1%) to the W-20-17 basal medium described above. W-20-17 cells were plated at 1.0 × 10^5^ cells/well in 200 μL assay medium in a 96-well tissue culture-treated polystyrene plate. rhBMP-2 was diluted as described above. Medium was removed from the monolayer of W-20-17 cells, and 200 μL of the assay media containing rhBMP-2 was added to each well. Assay medium containing no rhBMP-2 was used as the background sample. Cells were incubated in the presence of rhBMP-2 for 24 ± 4 h unless otherwise noted. Takedown of the plates and development were performed as described above.

### Modifications to standard protocols

1. To evaluate the dose response at higher concentrations, rhBMP-2 stocks were diluted to 4096 ng/mL, then diluted twofold to achieve a final concentration range from 1 to 2048 ng/mL.
2. To determine the effect of incubation time on the dose response, incubation times of 24 h, 3 days, and 6 days were tested.

### Analysis of data

The background signal from the samples containing no rhBMP-2 was subtracted from the absorbance value of each test sample. The bioactivity of the sample was determined by fitting the data using a four-parameter logistic equation model, where

*Y* = measured optical density at 405 nm,

*X* = concentration of rhBMP-2,

*A* = minimum optical density,

*B* = slope at the inflection point,

*C* = ED50 (concentration that reflects that at half of the maximum concentration), and

*D* = maximum optical density.

MATLAB software (version R2016b; Mathworks) was used for the curve fitting using a modified program.^*^ The upper limits of parameters A, B, C, and D were set at 1, infinity, 1369, and infinity, respectively. Lower limits were not set.

### Storage stability

To examine a correlation between the concentration and bioactivity of rhBMP-2, a 1 μg/mL solution of CHO-derived rhBMP-2 and *E.coli*-derived rhBMP-2 (E3) was prepared in the assay medium of W-20-17 cells; the stock solution was divided into 500 μL aliquots in centrifuge tubes. The aliquots were incubated at 37°C, 5% CO_2_, and RH 75%. At 6 h, 12 h, days 1, 2, 3, 5, 7, and 14, three aliquots were collected and stored at −80°C before enzyme-linked immunosorbent assay (ELISA; PeproTech) and bioactivity assay. The quantified concentrations from ELISA at each time point were normalized to those of day 0. The bioactivity of rhBMP-2 at each time point was measured following the procedure from ASTM Standard (F2131-02) with minor modifications, twofold dilutions were performed on each aliquot and incubated with W-20-17 cells directly for 24 h.

To evaluate the stability of rhBMP-2 at physiological temperature, a 100 ng/mL solution, as calculated using BCA assay, of each rhBMP-2 was prepared in the assay media of W-20-17 cells. The stock solution was then aliquoted into 500 μL aliquots in centrifuge tubes. The aliquots were incubated at 37°C, 5% CO_2_, and RH 75%. At days 1, 2, 3, 5, 7, 14, 21, and 28, three aliquots were collected for each rhBMP-2. The samples were stored at −80°C before ELISA (PeproTech). The quantified concentrations at each time point were normalized to those of day 0.

### Statistical analysis

Single-factor analysis of variance was performed followed by a multiple comparison *post-hoc* test (Dunnett's test) with an established significance of *p* ≤ 0.05. Data were reported as mean ± standard error. All statistical analyses were carried out in GraphPad Prism 7 software.

## Results

### RhBMP-2 dose response of model cell lines

**FIG. 1.**
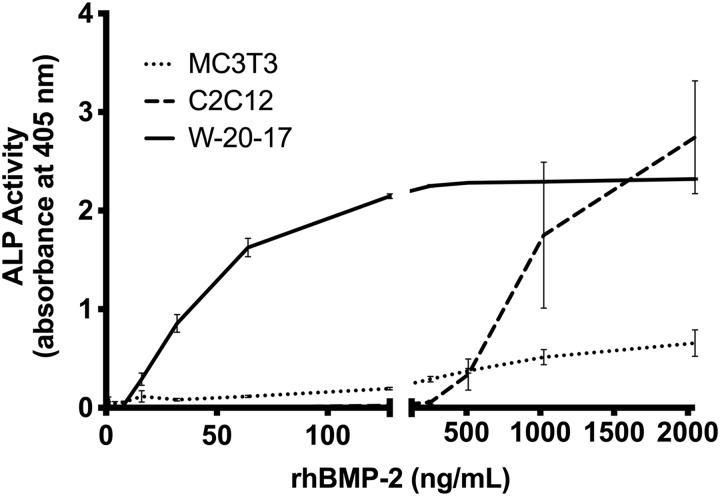
Dose response of rhBMP-2 in different model cell lines. The activity of rhBMP-2 (obtained from ECUST) was measured in C2C12 (*dashed*), MC3T3 (*dotted*), or W-20-17 cells (*solid*) after 72 h (3 days). Dose ranges from 1 to 2048 ng/mL with twofold dilutions were tested in all three cell lines. Data presented as average ± SE (*n* = 4). ECUST, East China University of Science and Technology; rhBMP-2, recombinant human bone morphogenetic protein-2; SE, standard error.

The induction of ALP in W-20-17, C2C12, and MC3T3 cells was compared as a function of the concentration of an *E. coli-*derived rhBMP-2 obtained from ECUST. After 72 h of incubation with a concentration range of 1–2048 ng/mL of rhBMP-2, ALP induction was measured with PNPP as a substrate (Fig. 1). C2C12 cells have a detection limit of ∼200 ng/mL, and show a strong dose response at concentrations >500 ng/mL and up to the highest concentration of rhBMP-2 used in this study. MC3T3 cells have a lower detection limit at ∼60 ng/mL, but the MC3T3 cell-based assay is less sensitive to variations in rhBMP-2 concentration and may therefore be a less desirable assay when accurate quantification is required. W-20-17 cells show the greatest sensitivity, with a detection limit at 10 ng/mL. A sharp dose-dependent response is observed up to 100 ng/mL, after which the signal reached saturation.

### Effect of incubation time on rhBMP-2 dose response

**FIG. 2.**
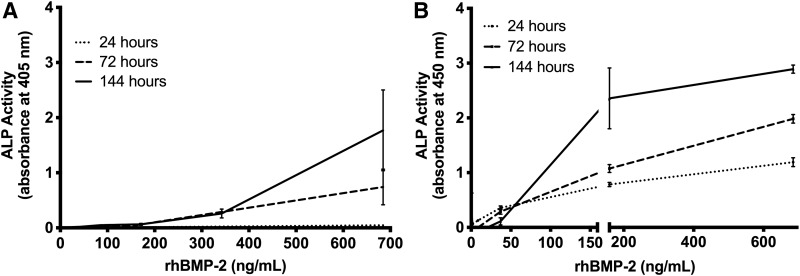
Effect of incubation time on rhBMP-2 dose response. rhBMP-2 activity was evaluated at the indicated period of time in C2C12 cells **(A)** and W-20-17 cells **(B)** for a low concentration range of 0–684.5 ng/mL of rhBMP-2 obtained from ECUST. Data presented as average ± SE (*n* = 4).

Incubation time with rhBMP-2 is another variable reported in the literature, with many studies reporting incubation times ranging from 1 to 7 days.^15–18^ The kinetics of ALP expression in response to rhBMP-2 induction may be different in different cell lines. To evaluate the effect of incubation time on the response to rhBMP-2, we compared the dose-dependent responses of C2C12 and W-20-17 cells to the same *E. coli*-derived rhBMP-2 as used before using a lower range of concentrations from 0 to 684.5 ng/mL (Fig. 2). As shown in Figure 2A, 24 h of incubation with rhBMP-2 resulted in little ALP activity in the C2C12 cell line. Increasing the incubation time to 3 and 6 days increased the response at the highest concentrations (684.5 ng/mL), but did not improve the sensitivity of the cell line at lower concentrations.

A different effect of incubation time was observed for W-20-17 cells. A 24-h incubation with the same low concentration range of rhBMP-2 increased the sensitivity of the W-20-17 cell line, as the detection limit was reduced to ∼2 ng/mL (Fig. 2B). The slope of the linear response range decreased, making the cell line more responsive over a wider range of concentrations but less sensitive to small differences in concentration. As incubation time increased to 3 and 6 days, the detection limit increased, but the slope of the linear range also increased, indicating an even narrower range of sensitivity.

### Comparison of commercially available rhBMP-2

**FIG. 3.**
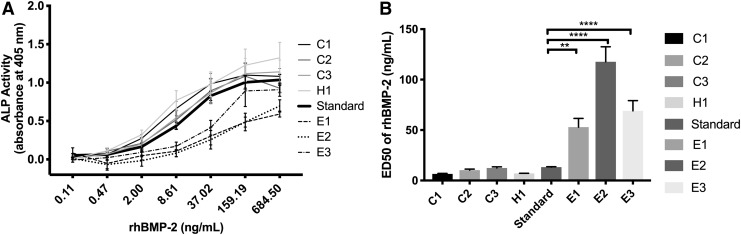
Bioactivity comparison of commercially available rhBMP-2. **(A)** RhBMP-2 dose–response curves obtained by incubating W-17-20 cells with rhBMP-2-containing medium for 24 h at the concentration range of 0.1–684.5 ng/mL. The international rhBMP-2 standard is indicated by the bolded line. **(B)** Quantification of ED50 values based on (A). Data presented as average ± SE (*n* = 3). One-way analysis of variance was performed on ED50 data, **p < 0.01, ****p < 0.0001.

We next used the W-20-17 cell line to compare the bioactivity of rhBMP-2 obtained from different commercial sources, CHO-derived rhBMP-2 (C1, C2, C3), *E. coli*-derived rhBMP-2 (E1, E2, E3), and HEK-293-derived rhBMP-2 (H1) (Fig. 3). As shown in Figure 3A, all tested samples of *E. coli*-derived rhBMP-2 triggered lower levels of ALP expression as compared with all of the samples of mammalian-derived rhBMP-2. This is also reflected by the ED50 values (Fig. 3B) where higher values indicate lower bioactivity. These results confirm the common observation that mammalian cell-derived rhBMP-2 is more active *in vitro* than *E. coli*-derived forms of rhBMP-2. The most bioactive preparations among our test samples were C1 and H1, but the differences among all mammalian cell-derived rhBMP-2 samples were not statistically significant.

### Storage stability

**FIG. 4.**
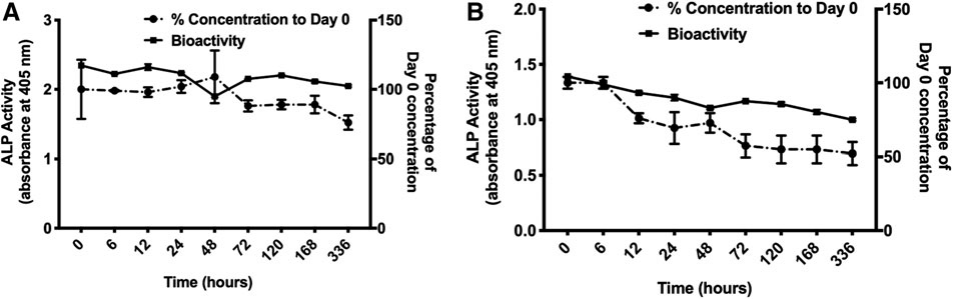
Stability of rhBMP-2 at 37°C over time. Concentration of rhBMP-2 in the medium was quantified using ELISA (*dashed*), and bioactivity by ALP induction was measured in response to rhBMP-2 stimulation (*solid*). CHO-derived rhBMP-2 (C1) **(A)** and *Escherichia coli-*derived rhBMP-2 (E3) **(B)** were tested using W-20-17 cells. Data presented as average ± SE (*n* = 3). ALP, alkaline phosphatase; CHO, Chinese hamster ovary; ELISA, enzyme-linked immunosorbent assay.

To define a correlation between the bioactivity of rhBMP-2 protein and protein concentration, a CHO-derived rhBMP-2 (C1) (Fig. 4A) and *E. coli*-derived rhBMP-2 (E3) (Fig. 4B) solution (1 μg/mL) were incubated in the assay medium of W-20-17 cells at 37°C for up to 14 days. Both bioactivity and protein concentration in the assay medium were measured at different time points (Fig. 4). For C1 (Fig. 4A) and E3 (Fig. 4B), the decrease of bioactivity followed the decrease in rhBMP-2 concentration. These results indicate that the change of rhBMP-2 concentration can be used as an approximate indicator of a change in bioactivity. This may be useful in some experimental designs since it is easier to determine rhBMP-2 concentration by ELISA than bioactivity by ALP expression.

**FIG. 5.**
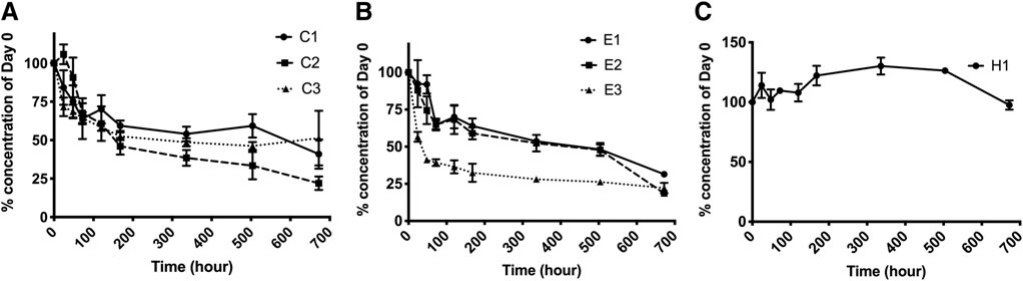
Stability of commercially available rhBMP-2 at 37°C. Concentration of CHO cell-derived rhBMP-2 **(A)**, *E. coli*-derived rhBMP-2 **(B),** and HEK-derived rhBMP-2 **(C)** at the indicated time points. The concentration was quantified by ELISA and normalized to that of day 0. Data are presented as average ± SE (*n* = 3). HEK, human embryonic kidney.

To determine the storage stability of different rhBMP-2 preparations, the concentrations of the different rhBMP-2 proteins in solution were measured by ELISA over time (Fig. 5). We expected that the ELISA-measured concentrations of rhBMP-2 would decrease over time. This basic behavior was indeed observed for all tested samples with the exception of the HEK-derived rhBMP-2 (H1), which showed an unexpected degree of stability throughout the incubation time (Fig. 5C). Figure 5A and B show sample-specific variations in storage stability at 37°C. There seems to be a general trend that the half-life of *E. coli*-derived BMP-2 (except for E3) was longer compared with CHO-derived BMP-2 (Fig. 5B). However, we did not find generally applicable correlations between storage stability and the source of rhBMP-2, indicating that the storage stability of any given sample of rhBMP-2 needs to be verified.

## Discussion

Since rhBMP-2 is expressed in different biological systems and may come from different manufacturing processes, its bioactivity has to be measured and reported to facilitate the comparison of results from different laboratories. Further, for comparative studies, a generally accepted bioactivity assay (used at a uniform concentration of rhBMP-2) is critically needed. Here, we report, for the first time, a direct comparison of different bioactivity assay parameters and an evaluation of the advantages and disadvantages of each assay. In addition, we compare the bioactivity of commercially available preparations of rhBMP-2 with an international standard, and assess the effect of glycosylation on the stability of rhBMP-2s during incubation under physiological conditions.

Currently, in the literature, different cell lines (i.e., C2C12, W-20-17, and MC3T3) and varying assay incubation times have been used to measure bioactivity of rhBMP-2. The varying responses from the three different model cell lines (Fig. 1) highlight the importance of choosing the proper cell line when evaluating rhBMP-2 bioactivity *in vitro*. Each cell line analyzed in this study shows different rhBMP-2 response kinetics, making them suitable for different applications. The ASTM Standard (F2131-02) uses the W-20-17 mouse stromal cell line, which has a very low detection limit of 2 ng/mL. This assay is best when rhBMP-2 is present only in low concentrations.^10,13,14^ C2C12 cells show little response at concentrations <200 ng/mL in agreement with previous reports,^1,15–18^ but a much greater response at higher concentrations (>500 ng/mL), a range at which the W-20-17 cells have already saturated in their signal output.

C2C12 cells express constitutively high levels of Msx2, which suppresses the mRNA and enzymatic activity levels of ALP induced by rhBMP-2.^22^ This explains why these cells require higher concentrations of rhBMP-2 to overcome the Msx2 suppression of ALP activity. As a myoblast cell line, the use of these cells to evaluate rhBMP-2 bioactivity can give key insight into the consequences of using rhBMP-2 above a certain concentration *in vivo*. The MC3T3 cells express a higher basal level of ALP activity compared with C2C12 and W-20-17 cells, and ALP is not upregulated to the same extent upon induction with rhBMP-2. However, the range over which the cells respond is much broader than either the C2C12 or W-20-17 cells, making it a better cell line to use if studying systems that take advantage of a broad concentration range.

After choosing the W-20-17 cell line as the model cell line of choice and a 24-h incubation as the incubation time, we validated the method using rhBMP-2 from commercial sources. The comparison of the commercially available BMP-2s with an international standard highlights the importance of the expression system used to produce the recombinant protein. The mammalian cell-derived rhBMP-2 had consistently lower ED50 values (higher bioactivity) than the *E. coli*-derived rhBMP-2.

The glycosylated form of the protein produced in mammalian cells is more hydrophilic, and the post-translational modification plays an important role in the recognition of the protein by the cells.^23,24^ On the contrary, it is worth noting that some studies demonstrate that *E. coli-*derived rhBMP-2 is able to trigger higher healing *in vivo*. This is probably because the lack of glycosylation increases the retention of *E. coli-*derived rhBMP-2 within the implanted carrier *in vivo* compared with that of the CHO-derived protein.^25^

We demonstrated that there is a correlation between rhBMP-2 bioactivity measured by ALP induction and rhBMP-2 concentration measured by ELISA. Therefore, the convenient measurement of rhBMP-2 concentration by ELISA can be used to obtain an estimate of the bioactivity of the protein, assuming that rhBMP-2 is losing its bioactivity through denaturation or degradation that will render the epitopes unrecognizable by the antibodies of an ELISA.

## Conclusion

We determined the different dose-responsive behaviors of W-20-17, MC3T3, and C2C12 cell lines. Among those cell lines, W-20-17 cells used as described in ASTM Standard (F2131-02) had the lowest limit of detection with a desirable dose-dependent response when low concentrations of rhBMP-2 are present. C2C12 cells have a detection limit of ∼200 ng/mL, and show a strong dose response at concentrations >500 ng/mL and up to ∼2000 ng/mL, the highest concentration of rhBMP-2 used in our study.

A bioactivity assay using C2C12 cells may be ideal when rhBMP-2 is present in higher concentrations and could be used, for example, as a quality test during the production of rhBMP-2 batches. Bioactivity assays using MC3T3 cells have a detection limit at ∼60 ng/mL, but the MC3T3 cell-based assay is less sensitive to variations in rhBMP-2 concentration. On the contrary, incubation time is an important variable in all cell-based bioactivity assays. Assays based on C2C12 cells gave optimal results at long incubation times of up to 6 days, while assays using W-20-17 cells performed well for all incubation times ranging from 24 h to 6 days.

We observed that rhBMP-2 generated from mammalian cells showed overall higher bioactivity than that from *E. coli*. Moreover, we established a relationship between bioactivity as measured by ALP induction and rhBMP-2 concentration as measured by ELISA. Therefore, concentration of rhBMP-2 being released from a delivery vehicle within the cellular dose-responsive range can be used as an indicator of rhBMP-2 bioactivity. Our results provide guidance for the establishment of suitable assays for the measurement of rhBMP-2 bioactivity under a wide range of experimental conditions.

## Notes

^*^ Cardillo G. Four parameters logistic regression—There and back again. 2012. https://it.mathworks.com/matlabcentral/fileexchange/38122
